# Assessment of whole-body and regional body fat using abdominal quantitative computed tomography in Chinese women and men

**DOI:** 10.1186/s12944-024-02034-y

**Published:** 2024-02-14

**Authors:** Jinci Mai, Qiulian Wu, Huanhua Wu, Chunyuan Zeng, Yingxin Li, Jingjie Shang, Biao Wu, Qijun Cai, Junbi Du, Jian Gong

**Affiliations:** 1 Department of Nuclear Medicine, First Affiliated Hospital, Jinan University, Guangzhou, China; 2 School of Nursing, Jinan University, Guangzhou, China; 3 Department of Clinical Medicine, International College, Jinan University, Guangzhou, China

**Keywords:** Quantitative computed tomography, Dual-energy X-ray absorptiometry, Total body fat percentage, Android, Gynoid

## Abstract

**Background:**

Being overweight or obese has become a serious public health concern, and accurate assessment of body composition is particularly important. More precise indicators of body fat composition include visceral adipose tissue (VAT) mass and total body fat percentage (TBF%). Study objectives included examining the relationships between abdominal fat mass, measured by quantitative computed tomography (QCT), and the whole-body and regional fat masses, measured by dual energy X-ray absorptiometry (DXA), as well as to derive equations for the prediction of TBF% using data obtained from multiple QCT slices.

**Methods:**

Whole-body and regional fat percentage were quantified using DXA in Chinese males (*n* = 68) and females (*n* = 71) between the ages of 24 and 88. All the participants also underwent abdominal QCT measurement, and their VAT mass and visceral fat volume (VFV) were assessed using QCT and DXA, respectively.

**Results:**

DXA-derived TBF% closely correlated with QCT abdominal fat percentage (*r* = 0.89–0.93 in men and 0.76–0.88 in women). Stepwise regression showed that single-slice QCT data were the best predictors of DXA-derived TBF%, DXA android fat percentage and DXA gynoid fat percentage. Cross-validation analysis showed that TBF% and android fat percentage could be accurately predicted using QCT data in both sexes. There were close correlations between QCT-derived and DXA-derived VFV (*r* = 0.97 in men and 0.93 in women).

**Conclusion:**

Clinicians can assess the TBF% and android and gynoid fat percentages of Chinese women and men by analysing existing abdominal CT-derived data using the QCT technique.

**Supplementary Information:**

The online version contains supplementary material available at 10.1186/s12944-024-02034-y.

## Background

In many nations, being overweight or obese has emerged as the main public health concern. The estimated numbers of adults with overweight or obesity in 2015 were 1.9 billion and 609 million, respectively, together representing approximately one-third of the global populace [1]. In China, about half of the adult population is overweight or obese [2]. Obesity is linked to an increased risk of diabetes, hypertension, metabolic syndrome and cardiovascular disease [3].

Several previous research has shown that high total body fat percentage (TBF%) is a separate risk indicator for type 2 diabetes and cardiovascular disease, whereas body mass index (BMI) is not [4, 5]. A reliable method to evaluate body composition is by dual energy X-ray absorptiometry (DXA), which assesses bone mineral density (BMD), muscle mass, adipose mass, and TBF% [6]. Quantitative computed tomography (QCT) is the quantitative analysis of computed tomography (CT) images using specialised software and specific calibration materials [7] and is considered to be a superior means of evaluating bone and fat distribution, because it provides real volumetric data for BMD, visceral adipose tissue (VAT) volume, subcutaneous adipose tissue (SAT) volume and total adipose tissue (TAT) volume [8, 9]. QCT technology can be used to analyse existing CT data obtained for other reasons, for instance, lung cancer screening, to assess the body composition across the scanned region without the need of patient time, additional equipment, radiation exposure, or significant extra costs. In a previous study, adolescent white girls' whole-body fat mass was estimated using peripheral quantitative computed tomography (pQCT) [10]. However, a literature search suggested that the use of abdominal QCT data for the prediction of TBF% has not previously been studied.

There are far more sets of CT equipment in China than DXA devices [11]; therefore, the rational use of BMD measurement and body composition analysis using low-dose CT has been an important area of research [12]. In addition, regional body composition analysis can be performed using a single low-dose CT scan, such as that used for screening for lung cancer or abdominal diseases. Under these circumstances, additional equipment, time, radiation exposure, and expenditure are not required [13]. However, performing whole-body CT scans to calculate TBF% is not feasible, because of the excessive radiation exposure associated.

A previous study showed that spinal and femoral DXA measurements could be used to accurately estimate TBF% [14]. Another study showed that single-slice QCT data are good predictors of visceral fat volume (VFV) in both sexes [3]. Therefore, the hypothesis of this study was that QCT data obtained using abdominal CT images could provide additional information regarding the abdominal fat content of patients and help clinicians evaluate the level of adiposity of patients. The following were the study's objectives: (1) to evaluate the correlation between TBF% derived from whole-body DXA scans and the abdominal fat percentage derived from multiple-slice QCT; (2) to identify the optimal QCT slice for the prediction of TBF% from the abdominal fat percentage; and (3) to compare the VAT data obtained using DXA and QCT.

## Methods

### Participants and exclusion criteria

It was a retrospective study of 68 men and 71 women of between ages of 24 and 88 who were all Chinese. The participants received positron emission tomography/ computed tomography (PET/CT) examination and were randomly assigned to Eq. (50 men and 50 women) and validation (18 men and 21 women) groups using SPSS software v.24.0 (IBM, Inc., Armonk, NY, USA). Individuals were excluded if they had: (1) a metal implant in their lumbar spine; (2) taken hormones or drugs that might alter body fat distribution, such as prednisone or growth hormone; or (3) a metabolic disease, such as type 2 diabetes, Down syndrome or Cushing’s syndrome. The study protocol was approved by the institutional Ethics Committee, and informed consent was acquired from all subjects involved.

### Anthropometric data

Body mass was measured using a platform digital scale to 0.1 kg and height was measured using a stadiometer to 0.5 cm while the participants were wearing light clothing and no shoes. BMI was calculated as body mass divided by height squared (kg/m^2^). All the participants underwent QCT scans and DXA body composition examinations. To eliminated the influence of radionuclides on the DXA examination, both examinations were performed within 1 month, with an interval of > 3 days between them [15, 16].

### DXA measurements

DXA images (GE Lunar iDXA, GE Healthcare, Madison, WI, USA) were analysed using enCORE software (ver. 16.0, GE Medical Systems, Madison, WI, USA). All the participants were scanned in the position recommended by the International Society for Clinical Densitometry, with their upper limbs lying flat along their body, their palms down and not overlapping their body, their feet in a neutral or slightly internally rotated position, their head and chin positioned centrally and face up [17]. A whole-body scan could obtain their android and gynoid fat percentages. The android region of interest (ROI) was defined as the sovra-umbilical abdominal region, with an upper boundary of a horizontal line drawn twenty percent of the way between the pelvis and the head, lateral boundaries of the margins of the trunk, and a lower boundary at the pelvis [18]. The gynoid ROI was below the android ROI and represented the gluteofemoral region. Its upper boundary was placed at 1.5-times the distances of the android ROI from the pelvic line, bilateral hip lines were used and the lower boundary was placed at twice the distances of the android ROI [18]. VAT mass and VFV were obtained using the “CoreScan” module of the enCORE software by subtracting the fat mass from the TAT in the android ROI on both sides of the abdominal cavity [19]. The software produced each of these ROIs on its own. Before operation, the DXA scanner underwent a once-daily quality control evaluation. The scans and analysis were completed and evaluated by the same professional technician. For 30 of the participants, the precision errors were < 2% for the total fat mass and < 3% for the regional fat mass.

### QCT measurements

The same PET/CT scanner (Discovery 690, GE Healthcare) was used to generate cross-sectional CT images of the abdomen of each participant from the twelfth thoracic vertebra to the first sacral vertebra (T12 to S1). The parameters used were 120 kV, 200 mA and a 1.25-mm slice thickness. A technician calibrated the machine using a phantom once a month for quality control purposes. The abdominal adipose tissue areas across multiple slices at the levels of the T12/L1, L1/L2, L2/L3, L3/L4, L4/L5, and L5/S1 intervertebral discs and the distances between the adjacent levels were measured using Mindways QCT Pro (Mindways 6.1, Austin, TX, USA) software. This software automatically set closed snake splines at the boundaries between the subcutaneous fat and abdominal muscle, and between the VAT and SAT [20], and then calculated the VAT mass and visceral fat area at the above six levels. The values for each parameter at each level multiplied by the distances between the adjacent levels were used to calculate the VAT mass and VFV [21]. The percentage of fat in each slice was used to calculate the abdominal fat percentage. All the participants underwent CT scans from T12 to S1 that were performed by a trained QCT technician. This analysis did not involve any further radiation exposure.

### Statistical analyses

Statistical analyses were performed using SPSS and MedCalc Statistical Software version 15.2.2 (MedCalc Software bvba, Ostend, Belgium). Normally distributed data are expressed as mean and standard deviation (SD) and non-normally distributed data are expressed as median and inter-quartile range (IQR). One-way ANOVA, the Kruskal–Wasllis test and post hoc analysis were employed to contrast the participants' baseline characteristics in the equation and validation groups, as appropriate. Pearson’s correlation coefficient (r), Spearman’s correlation coefficient (r) and the concordance correlation coefficient were used to evaluate the relationships between the data obtained using the two devices, as appropriate. Predictive equations were generated by stepwise regression using data collected from the equation group, in which age, height, weight and BMI were regarded as covariates. Regression analysis and Bland–Altman analysis were performed on validation group data to assess the accuracy of these equations. There was a significance level of 0.05 and all tests were two-sided.

## Results

### Characteristics of the participants

**Fig. 1:**
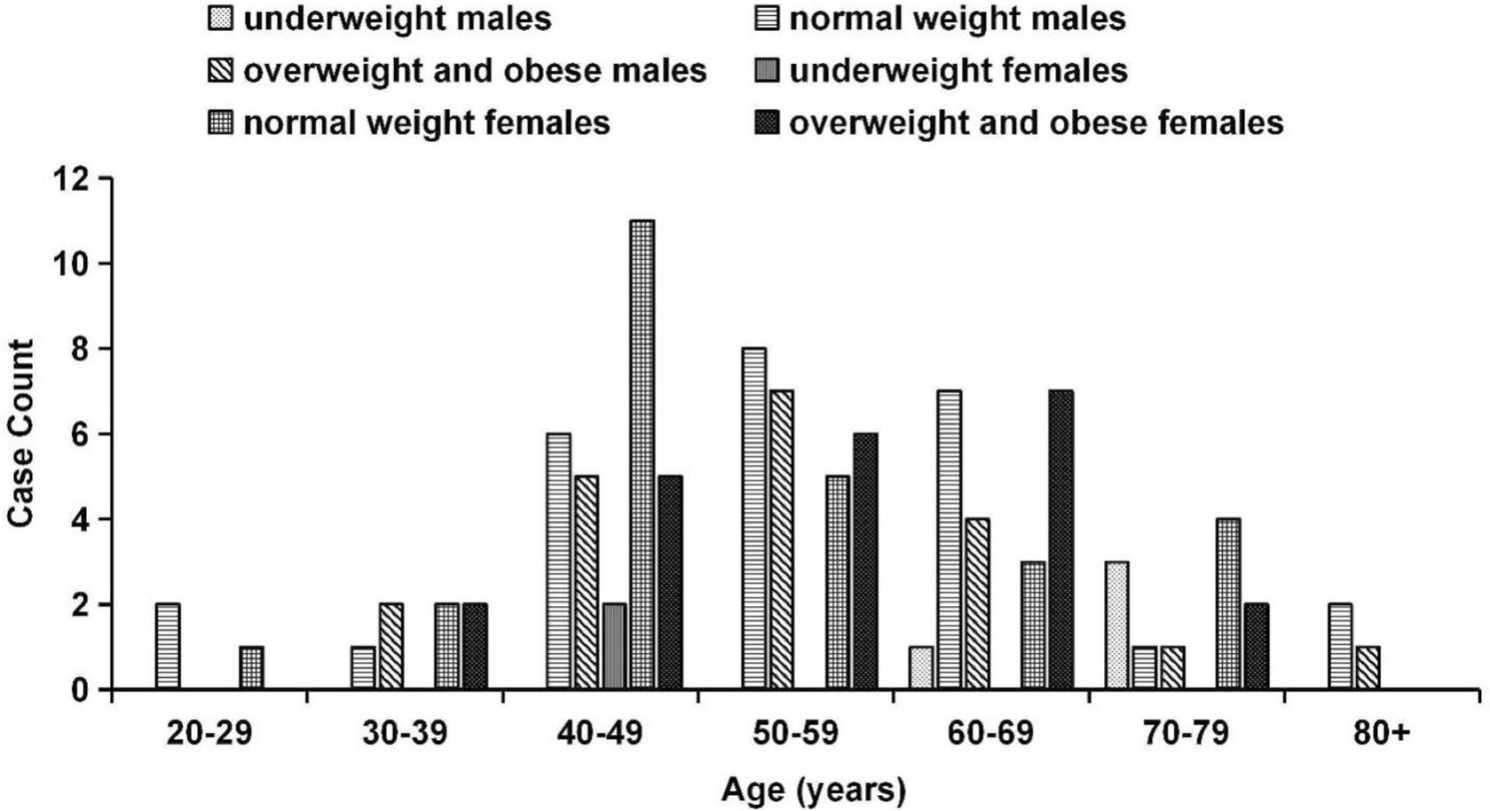
The distributions of age and BMI among the 50 male and 50 female participants in the equation group. Underweight: < 18 kg/cm^2^; normal weight: BMI 18–23.9 kg/cm^2^; overweight and obesity: BMI ≥ 24 kg/cm^2^. BMI, body mass index

Table 1 lists the physical characteristics and body compositions of the equation and validation groups. The age and BMI distribution of the participants used to generate the equations are shown in Fig. 1. The male participants were significantly taller than the female participants in the equation group, and all the DXA-derived and QCT-derived fat percentages were significantly lower, except at the T12/L1 and L1/L2 levels. The DXA-derived and QCT-derived VFVs in the equation group exhibited a close correlation (*r* = 0.95, *P* < 0.001). The male participants in the equation group were significantly taller and heavier than the female participants in the validation group, and the TBF%, gynoid fat percentages, L3/L4%fat and L5/S1%fat were significantly lower. The male participants in the validation group were significantly taller than the female participants in the equation group, and all the DXA-derived and QCT-derived fat percentages were significantly lower, except at the T12/L1 to L2/L3 levels. The male participants were significantly taller and heavier than the female participants in the validation group, and the TBF%, gynoid fat percentages and L5/S1%fat were significantly lower. The other characteristics or body composition of the two sexes of participants did not have difference.

**Table 1:** Physical characteristics and body composition of the participants, categorised according to sex

	Equation group		Validation group	
	Males (*n* = 50)	Females (*n* = 50)	Males (*n* = 18)	Females (*n* = 21)
	Mean ± SD/ Median(IQR)	Mean ± SD/ Median(IQR)	Mean ± SD/ Median(IQR)	Mean ± SD/ Median(IQR)
Age (yr)	55.8 ± 13.3	52.6 ± 11.6	57.3 ± 9.9	59.3 ± 13.3
Anthropometric measures				
Height (cm)	167.1 ± 5.7	157.8 ± 4.9^a^	165.8 ± 9.7^b^	155.2 ± 6.4^ac^
Weight (kg)	63.3 ± 10.2	55.0 (50.0–68.0)	63.0 ± 13.6	52.3 ± 8.8^ac^
BMI (kg/m^2^)	22.6 ± 2.9	23.4 ± 3.7	22.8 ± 3.3	21.6 ± 3.1
DXA measures				
DXA VAT mass (g)	940.7 ± 535.7	621.3(375.4–1118.6)	925.6 ± 532.4	691 ± 377.7
DXA VFV (cm^3^)	977.1 ± 567.8	658.6(398.0–1185.8)	981.1 ± 564.3	732.4 ± 400.4
TBF%	24.9 ± 6.2	35.5 ± 6.3^a^	24.0 ± 7.3^b^	32.4 ± 7.0^ac^
Android %fat	31.0 ± 10.7	40.4 ± 9.1^a^	29.7 ± 12.2^b^	36.5 ± 11.8
Gynoid %fat	24.3 ± 5.0	38.5 ± 5.8^a^	23.7 ± 6.4^b^	34.1(32.9–37.3)^ac^
QCT measures				
QCT VAT mass (g)	647.2(340.1–877.7)	505.5(325.6–688.3)	611.5 ± 311.2	432.0 ± 189.1
QCT VFV (cm^3^)	2046.6 ± 972.3	1629.3(1236.0–2427.6)	2090.1 ± 1070.3	1572.4 ± 654.4
T12/L1%fat	29.2(12.0–43.9)	33.3 ± 15.5	30.0 ± 17.3	32.5 ± 14.6
L1/L2%fat	37.2(18.5–51.4)	42.7 ± 14.3	36.3 ± 18.4	40.4 ± 16.7
L2/L3%fat	45.5(33.6–52.2)	54.6(43.6–58.9)^a^	44.3(37.2–55.5)	51.2(42.7–56.8)
L3/L4%fat	48.3(38.6–54.7)	57.3(49.2–60.7)^a^	47.5(39.3–56.3)^b^	56.5(44.1–61.0)^a^
L4/L5%fat	49.7(42.5–56.2)	59.1 ± 8.1^a^	50.8(40.0–56.7)^b^	58.4(51.7–61.3)
L5/S1%fat	44.7(37.3–44.7)	57.6 ± 8.5^a^	43.2(36.7–51.2)^b^	55.0(46.9–59.6)^ac^

*BMI* body mass index, *DXA* dual-energy X-ray absorptiometry, *VAT* visceral adipose tissue, *VFV* visceral fat volume, *TBF%* total body fat percentage, *QCT* quantitative computed tomography, *SD* standard deviation, *IQR* interquartile range

^a^*P* < 0.05 compared to male participants in the equation group

^b^*P* < 0.05 compared to the female participants in the equation group

^c^*P* < 0.05 compared to male participants in the validation group

### Relationships between DXA and QCT data

The relationships among the DXA and QCT data obtained for participants of each sex in the equation group are displayed in Table 2. Significant positive correlations (*P* < 0.001) were observed in all subgroups between the DXA and QCT data. Substantial similarity was identified between VFV data generated using the two devices (*r* = 0.97 for men and 0.93 for women). There were closer correlations between the DXA and QCT data (fat percentage) in men than in women participants (*r* = 0.66–0.94 for men and 0.49–0.89 for women). TBF% closely correlated with L1/L2 fat percentage in men (*r* = 0.93), and with L5/S1 fat percentage in women (*r* = 0.88). The android fat percentage correlated most closely with the L3/L4 fat percentage in men (*r* = 0.94), and the L5/S1 fat percentage in women (*r* = 0.89); and the gynoid fat percentage correlated most closely with the L5/S1 fat percentage (*r* = 0.79 for men and 0.69 for women) in both sexes.

**Table 2:** Pearson’s or Spearman’s correlation coefficients (r) for VAT mass, VFV, and fat percentage assessed using DXA or QCT

	Males	Females
DXA VAT mass vs QCT VAT mass	0.92**	0.85**
DXA VFV vs QCT VFV	0.97**	0.93**
TBF% vs T12/L1%fat	0.90**	0.83**
TBF% vs L1/L2%fat	0.93**	0.76**
TBF% vs L2/L3%fat	0.91**	0.83**
TBF% vs L3/L4%fat	0.91**	0.84**
TBF% vs L4/L5%fat	0.89**	0.86**
TBF% vs L5/S1%fat	0.89**	0.88**
Android %fat vs T12/L1%fat	0.93**	0.82**
Android %fat vs L1/L2%fat	0.93**	0.79**
Android %fat vs L2/L3%fat	0.91**	0.84**
Android %fat vs L3/L4%fat	0.94**	0.87**
Android %fat vs L4/L5%fat	0.91**	0.88**
Android %fat vs L5/S1%fat	0.87**	0.89**
Gynoid %fat vs T12/L1%fat	0.66**	0.56*
Gynoid %fat vs L1/L2%fat	0.72**	0.49**
Gynoid %fat vs L2/L3%fat	0.72**	0.55**
Gynoid %fat vs L3/L4%fat	0.73**	0.56**
Gynoid %fat vs L4/L5%fat	0.73**	0.62**
Gynoid %fat vs L5/S1%fat	0.79**	0.69**

*DXA* dual-energy X-ray absorptiometry, *QCT* quantitative computed tomography, *VAT* visceral adipose tissue, *VFV* visceral fat volume, *TBF%* total body fat percentage

^*^P < 0.05, **P < 0.001. The bold data are Spearman’s correlation coefficients

### Outcomes of the stepwise regression analysis

Tables 3 and 4 present the outcome of a stepwise regression analysis of whole-body and regional fat percentages. In men, the L1/L2 fat percentage (*R*^2^ = 0.81) and L2/L3 fat percentage (*R*^2^ = 0.79) provided the best estimates of TBF%, and the L5/S1 fat percentage provided the best estimate of TBF% in women (*R*^2^ = 0.77). The introduction of BMI (1.6% and 4.1% in men) or body mass (4.4% in women) made small contributions to the prediction of TBF%. In the equations for predicting android fat percentage, the L1/L2 fat percentage was found to adequately explain most of the variation in android fat percentage in men (85.6%), and the L3/L4 fat percentage was found to explain 81.7% of the variation in android fat percentage in women. For gynoid fat percentage, moderately good predictions were obtained using the L5/S1 fat percentage in men (*R*^2^ = 0.65) and the L5/S1 fat percentage and age in women (*R*^2^ = 0.51).

**Table 3:** Results of the stepwise regression analysis for the prediction of whole-body and regional fat percentages in men in the equation group

Dependent DXA variable	Independent QCT variable	Prediction equation	Adjusted R^2^	SEE
TBF%	T12/L1%fat	0.27 × T12/L1%fat + 0.51 × BMI + 5.89	0.77	2.98
	L1/L2%fat	0.27 × L1/L2%fat + 0.43 × BMI + 5.76	0.83	2.55
	L2/L3%fat	0.28 × L2/L3%fat + 0.60 × BMI-0.29	0.83	2.56
	L3/L4%fat	0.44 × L3/L4%fat + 4.85	0.81	2.69
	L4/L5%fat	0.36 × L4/L5%fat + 0.50 × BMI-3.22	0.80	2.77
	L5/S1%fat	0.41 × L5/S1%fat + 0.60 × BMI-6.19	0.80	2.79
Android %fat	T12/L1%fat	0.46 × T12/L1%fat + 1.12 × BMI-7.42	0.87	3.90
	L1/L2%fat	0.43 × L1/L2%fat + 1.12 × BMI-9.49	0.90	3.43
	L2/L3%fat	0.45 × L2/L3%fat + 1.41 × BMI-19.34	0.89	3.50
	L3/L4%fat	0.63 × L3/L4%fat + 0.27 × weight-14.86	0.89	3.50
	L4/L5%fat	0.58 × L4/L5%fat + 1.22 × BMI-23.85	0.87	3.83
	L5/S1%fat	16.54 × log(L5/S1%fat) + 1.88 × BMI-73.14	0.80	4.82
Gynoid %fat	T12/L1%fat	0.13 × T12/L1%fat + 0.59 × BMI + 7.40	0.47	3.68
	L1/L2%fat	0.21 × L1/L2%fat + 16.90	0.52	3.50
	L2/L3%fat	0.18 × L2/L3%fat + 0.46 × BMI + 6.42	0.56	3.32
	L3/L4%fat	0.31 × L3/L4%fat + 10.29	0.59	3.23
	L4/L5%fat	0.31 × L4/L5%fat + 9.61	0.56	3.34
	L5/S1%fat	0.40 × L5/S1%fat + 6.91	0.65	2.98

*DXA* dual-energy X-ray absorptiometry, *TBF%* total body fat percentage, *QCT* quantitative computed tomography, *adjusted R*^*2*^ adjusted coefficient of determination, *SEE* standard error of the estimate

**Table 4:** Results of the stepwise regression analysis for the prediction of whole-body and regional fat percentages in women in the equation group

Dependent DXA variable	Independent QCT variable	Prediction equation	Adjusted R^2^	SEE
TBF%	T12/L1%fat	0.34 × T12/L1%fat + 24.36	0.68	3.54
	L1/L2%fat	0.22 × L1/L2%fat + 0.22 × weight + 12.37	0.64	3.74
	L2/L3%fat	0.26 × L2/L3%fat + 0.22 × weight + 9.62	0.71	3.36
	L3/L4%fat	0.38 × L3/L4%fat + 0.51 × BMI + 2.88	0.77	2.99
	L4/L5%fat	0.50 × L4/L5%fat + 0.57 × BMI-7.41	0.80	2.83
	L5/S1%fat	0.52 × L5/S1%fat + 0.16 × weight-3.52	0.81	2.73
Android %fat	T12/L1%fat	0.34 × T12/L1%fat + 0.75 × BMI + 11.51	0.69	5.09
	L1/L2%fat	0.34 × L1/L2%fat + 0.31 × weight + 7.92	0.68	5.17
	L2/L3%fat	0.47 × L2/L3%fat + 0.27 × BMI + 1.53	0.83	3.83
	L3/L4%fat	0.62 × L3/L4%fat + 0.66 × BMI-8.81	0.86	3.48
	L4/L5%fat	0.75 × L4/L5%fat + 0.84 × BMI-23.88	0.84	3.69
	L5/S1%fat	0.76 × L5/S1%fat + 0.23 × weight-17.14	0.83	3.82
Gynoid %fat	T12/L1%fat	0.21 × T12/L1%fat + 31.45	0.30	4.83
	L1/L2%fat	0.15 × L1/L2%fat + 0.47 × BMI-0.13 × age + 27.77	0.32	4.78
	L2/L3%fat	0.17 × L2/L3%fat + 0.52 × BMI-0.14 × age + 25.47	0.33	4.73
	L3/L4%fat	0.31 × L3/L4%fat + 21.76	0.31	4.81
	L4/L5%fat	0.50 × L4/L5%fat-0.13 × age + 15.80	0.42	4.39
	L5/S1%fat	0.51 × L5/S1%fat-0.12 × age + 15.08	0.51	4.04

*DXA* dual-energy X-ray absorptiometry, *TBF%* total body fat percentage, *QCT* quantitative computed tomography, *adjusted R*^*2*^ adjusted coefficient of determination, *SEE* standard error of the estimate

### Cross-validation

**Fig. 2:**
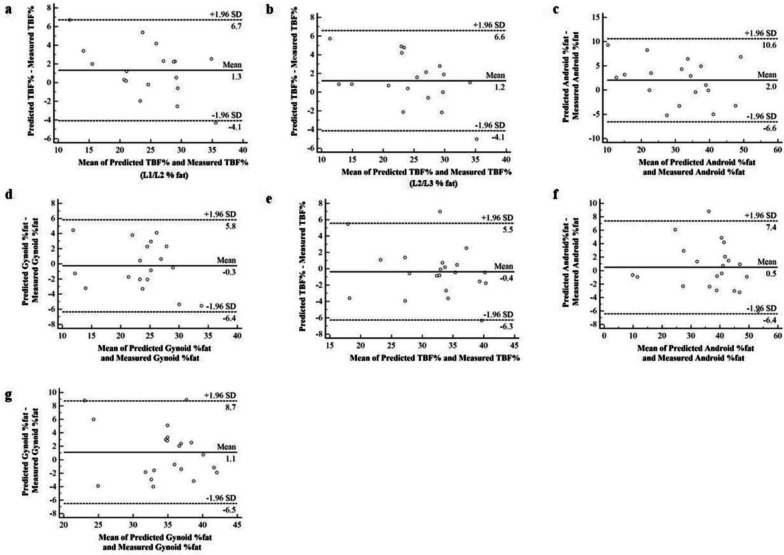
Bland–Altman plots used in the validation group for cross-validation. Dashed lines represent the limits of agreement (mean difference ± 1.96 SDs) and solid lines represent the mean difference between the predicted and measured values. **a**-**d**: Cross-validation of the predictive equations in men; **e**-**g**: cross-validation of the predictive equations in women. TBF%, total body fat percentage; SD, standard deviation

**Fig. 3:**
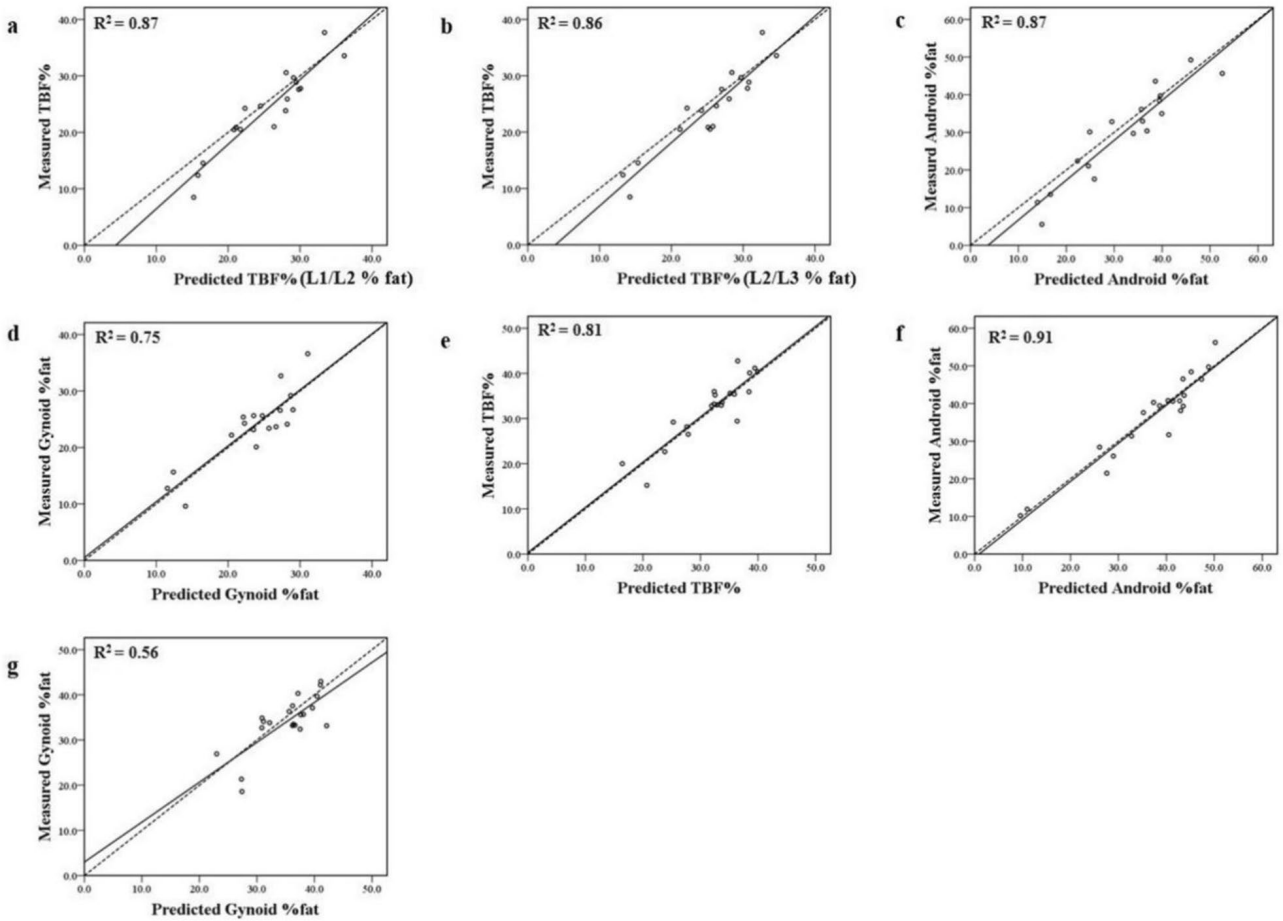
Scatterplots used in the validation group for cross-validation. Dashed lines represent the identity lines and solid lines represent the regression lines for the predicted and measured values. **a**-**d**: Cross-validation of the predictive equations in men; (e)–(g): cross-validation of the predictive equations in women. TBF%, total body fat percentage; R^2^, coefficient of determination

The equations generated using the data in Tables 3 and 4 were cross-validated using an independent sample of 39 participants. Table 5 displays the outcomes of the Bland–Altman and regression analyses. The *P*-values for the slopes were all < 0.05 on regression analysis. There was substantial agreement between the measured and anticipated values in the Bland–Altman plots (Fig. 2), in the absence of systematic bias (*P* > 0.05). The predictive equations for TBF% and gynoid fat percentage were more comparable for the men than for the women, but the opposite was true for the equations for the android fat percentage (Figs. 2 and 3). The comparability of the visceral fat data obtained using DXA and QCT was checked using the equation group, and the results showed that they were not particularly consistent (concordance correlation coefficient: 0.45–0.70) (Supplementary document).

**Table 5:** The overview of the prediction equations created in Tables 3 and 4 by cross-validation

Independent variable	Regression analysis				Bland–Altman	
	Intercept	Slope	Adjusted R^2^	SEE	Mean difference	95% LoA
Predicted TBF%						
Male (L1/L2)	-5.00	1.145	0.87	2.68	1.31	-4.08 to 6.71
Male (L2/L3)	-4.32	1.122	0.86	2.71	1.22	-4.14 to 6.58
Female (L5/S1)	0.33	1.002	0.81	3.08	-0.37	-6.29 to 5.54
Predicted Android %fat						
Male (L1/L2)	-3.90	1.060	0.87	4.48	2.00	-6.58 to 10.58
Female (L3/L4)	-0.95	1.013	0.91	3.62	0.47	-6.43 to 7.38
Predicted Gynoid %fat						
Male (L5/S1)	0.48	0.991	0.75	3.20	-0.27	-6.36 to 5.82
Female (L5/S1)	2.98	0.884	0.56	3.94	1.10	-6.51 to 8.72

*TBF%* total body fat percentage, *adjusted R*^*2*^ adjusted coefficient of determination, *SEE* standard error of the estimate, *LoA* limits of agreement

## Discussion

Recent studies of the methods used for the prediction of TBF% have mostly been based on measurements such as skin-fold thickness, limb lengths, waist circumference, hip circumference and BMI [22–24]. A study of 350 adult women established three equations for the prediction of TBF% based on anthropometric parameters, of which the prediction equation based on hip circumference and skin-fold thickness was the most accurate (*R*^2^ = 0.72) [22]. Another study of 64 adults established an equation for the prediction of TBF%, assessed using bioelectrical impedance analysis (BIA), based on neck circumference, conicity index, body shape index, waist-hip ratio and BMI, with an R^2^ of 0.839 [25]. However, these indices represent approximations of fat mass and are of relatively limited use for the prediction of TBF%. In contrast, Zhang et al. have shown that the DXA-derived spinal fat percentage is an effective predictor of DXA-derived TBF% [26]. Furthermore, Cheng et al*.* have previously shown that single-slice QCT data accurately predict VFV (*R*^2^ = 0.96) in Chinese people [3]. Therefore, it was hypothesised that QCT data would also be an effective means of predicting TBF%.

TBF% could be largely explained by abdominal fat percentage in the present study. The best predictors were found to be L5/S1 fat percentage with body mass in women (*R*^2^ = 0.81), and L1/L2 fat percentage with BMI (*R*^2^ = 0.83) and L2/L3 fat percentage with BMI (*R*^2^ = 0.83) in men. The cross-validation analysis showed that the accuracy of the predictive equations using L1/L2 fat percentage or L2/L3 fat percentage were also similar (SEE: 2.68, 95% LoA: − 4.08 to 6.71; SEE: 2.71, 95% LoA: − 4.14 to 6.58). Clinicians can choose the reference layer according to the region of the body that underwent CT examination. Although the accuracy of the generated predictive equations is similar to that of equations generated using anthropometric parameters, the R^2^ of the former is higher [22]. However, BIA overestimates the TBF% of people living with obesity, and therefore the results of studies that used BIA to assess TBF% may be biased [25, 27]. In the equation group, approximately 42% of the participants were overweight or obese (40% of the men and 44% of the women). The weight distribution of the participants was relatively balanced; therefore, it was believing that the predictive equations generated in the present study avoid such bias and are superior to the predictive equations generated using anthropometric parameters. Indeed, a single-slice abdominal fat percentage was found to be a good predictor, with the L1/L2 fat percentage explaining 81.4% of the variance in TBF% in men and the L5/S1 fat percentage explaining 76.6% of the variance in women. The differing results obtained for men and women may be explained by their differing patterns of fat accumulation. In general, men predominantly accumulate fat around the abdomen, whereas women often accumulate adipose tissue more distally [28, 29], and this is consistent with the findings that L1/L2 fat mass (android) and L5/S1 fat mass (gynoid) were superior predictors in men and women, respectively.

Previous research has demonstrated that the accumulation of body fat in the abdominal region, and especially in the android region, is more strongly linked to metabolic syndrome and cardiovascular disease than TBF% [30]. Thus, the goal of the current study was to predict the android and gynoid fat percentages using single-slice abdominal fat percentages. Interestingly, the correlations between L3/L4 fat percentage and android fat percentage and between L5/S1 fat percentage and gynoid fat percentage were the closest in both sexes. However, the regression model showed that android fat percentage was best estimated using L1/L2 fat percentage and BMI in men (*R*^2^ = 0.90), but using L3/L4 fat percentage and BMI in women (*R*^2^ = 0.86). The gynoid fat percentage was best estimated using the L5/S1 fat percentage alone in men (*R*^2^ = 0.65), and using the L5/S1 fat percentage and age in women (*R*^2^ = 0.51). Cross-validation of these predictive equations showed that they accurately estimated android fat percentage in both sexes and gynoid fat percentage in men, whereas the accuracy of the prediction of gynoid fat percentage was found to be poor in women. The reason behind this could be due to the redistribution of adipose tissue from the lower body to the abdomen during aging, which reduces the difference in android fat accumulation between the sexes [29]. Thus, because gynoid fat percentage is affected by age and sex, the predictive equations generated were unsatisfactory.

Disease like insulin resistance and metabolic-associated fatty liver disease are thought to be significantly increased by high VAT mass [31, 32]. The visceral adiposity index, an index for the evaluation of VAT, was found to be associated with aging in a study of 6,252 adults in the USA [33]. However, QCT is now thought to be a superior method for the assessment of VAT [9]. VAT is the total adipose tissue pixels from the linea alba inside the rectus abdominis, internal oblique, iliac, and peritoneal planes [34]. VAT mass is estimated using DXA by subtracting the subcutaneous fat on both sides from the TAT in the android region and multiplying by fat density (0.94 g/cm^3^) to yield the VFV [35]. In the present study, the QCT data were obtained from a single slice, whereas the DXA analysis data were obtained from the android region. Because some medical institutions do not have the ability to perform QCT, the “CoreScan” software was created to calculate VAT using DXA images. Previous studies have shown that VFV measured using DXA closely correlates with that assessed using CT (*r* = 0.83) and MRI (*r* = 0.90) [36]. Therefore, the VAT values obtained using DXA and QCT in the present study were compared and the correlation coefficients were found to be very close to 1.0 (*r* = 0.97 for men and 0.93 for women). However, the VAT values were found not to be particularly comparable in the consistency analysis, implying that the accuracy of VAT values assessed using DXA is questionable, and therefore its clinical utility should be further evaluated.

## Strengths and limitations

The present study had two main strengths. First, it is the first time to use fat parameters obtained from abdominal QCT for the prediction of whole-body and regional fat in Chinese people. Second, the results obtained using DXA and QCT, which are recognised to be quantitative means of evaluating human body composition, were compared. Only a few other studies have made such comparisons.

The present study also had several limitations. First, although researchers collected as many samples as possible, but how to obtain the appropriate sample size still needs further calculation. The sample size was relatively small, in addition, all of the participants came from southern China; therefore, the results are not fully representative of the Chinese population as a whole. Second, the data were not categorised according to the BMI of the participants, and therefore the predictive equations might be less accurate for people with certain BMIs. Third, the study's findings mostly demonstrate how accurate the equations are in predicting middle-aged adults because younger and older adults were underrepresentation. Finally, the participants were not randomly sampled healthy people; therefore, further cross-validation studies of healthy individuals performed by QCT using equipment supplied by other manufacturers should be performed.

## Conclusion

The current study's outcomes have been identified close correlations between TBF% estimated using DXA and QCT-derived abdominal fat percentages. The single-slice abdominal fat percentage and anthropometric data together were found to provide excellent estimates of TBF% and android fat percentage in Chinese men and women. In addition, gynoid fat percentage could be estimated accurately using single-slice abdominal fat percentages and BMI in Chinese men. Furthermore, for patients who undergo chest or abdominal CT scans for other purposes, clinicians can select slices that are useful for the prediction of TBF%, android fat percentage or gynoid fat percentage without the necessity for additional exposure to radiation. Using this approach, the radiation dose received by, and the expenditure of, patients can be reduced.

### Supplementary Information

**Additional file 1. **

**Additional file 2. **

**Additional file 3. **

**Additional file 4. **

## Data Availability

The datasets used and analysed during the study will be made available by the corresponding author upon reasonable request.

## Abbreviations

TBF%: Total body fat percentage
BMI: Body mass index
DXA: Dual energy X-ray absorptiometry
BMD: Bone mineral density
QCT: Quantitative computed tomography
CT: Computed tomography
VAT: Visceral adipose tissue
SAT: Subcutaneous adipose tissue
TAT: Total adipose tissue
pQCT: Peripheral quantitative computed tomography
VFV: Visceral fat volume
PET/CT: Positron emission tomography/ computed tomography
ROI: Region of interest
SD: Standard deviation
IQR: Inter-quartile range
BIA: Bioelectrical impedance analysis
