# Supplementary material for: Assessment of whole-body and regional body fat using abdominal quantitative computed tomography in Chinese women and men
Source: Lipids Health Dis. 2024 Feb 14;23:47. doi: 10.1186/s12944-024-02034-y (PMC10865662; doi:10.1186/s12944-024-02034-y)
Supplement: Supplementary file 1 — Additional file 1. [file 12944_2024_2034_MOESM1_ESM.docx]

Attached are some photos of the Chinese informed consent form, which are translated into English as follows:

Nuclear medicine department of the First Affiliated Hospital of Jinan University free DXA examination informed consent

Dear Sir (Madam)

According to your information, you meet the admission criteria of a DXA clinical trial study conducted by Nuclear medicine department of the First Affiliated Hospital of Jinan University. If you are willing to participate in this trial study, you can get a chance to measure bone mineral density and body composition for free. We will make a diagnosis of your bone mineral density and give relevant recommendations on bone health. The test will generate very small amount of X-rays, only about one-tenth of the amount of radiation of a chest X-ray, which is a very safe test and will not cause damage to your body.

If you agree to take part in this study, please fill in the agreement and sign, thank you!

Subject's signature: Doctor's signature:

Date: Date:

Nuclear Medicine Department of the First Affiliated Hospital of Jinan University


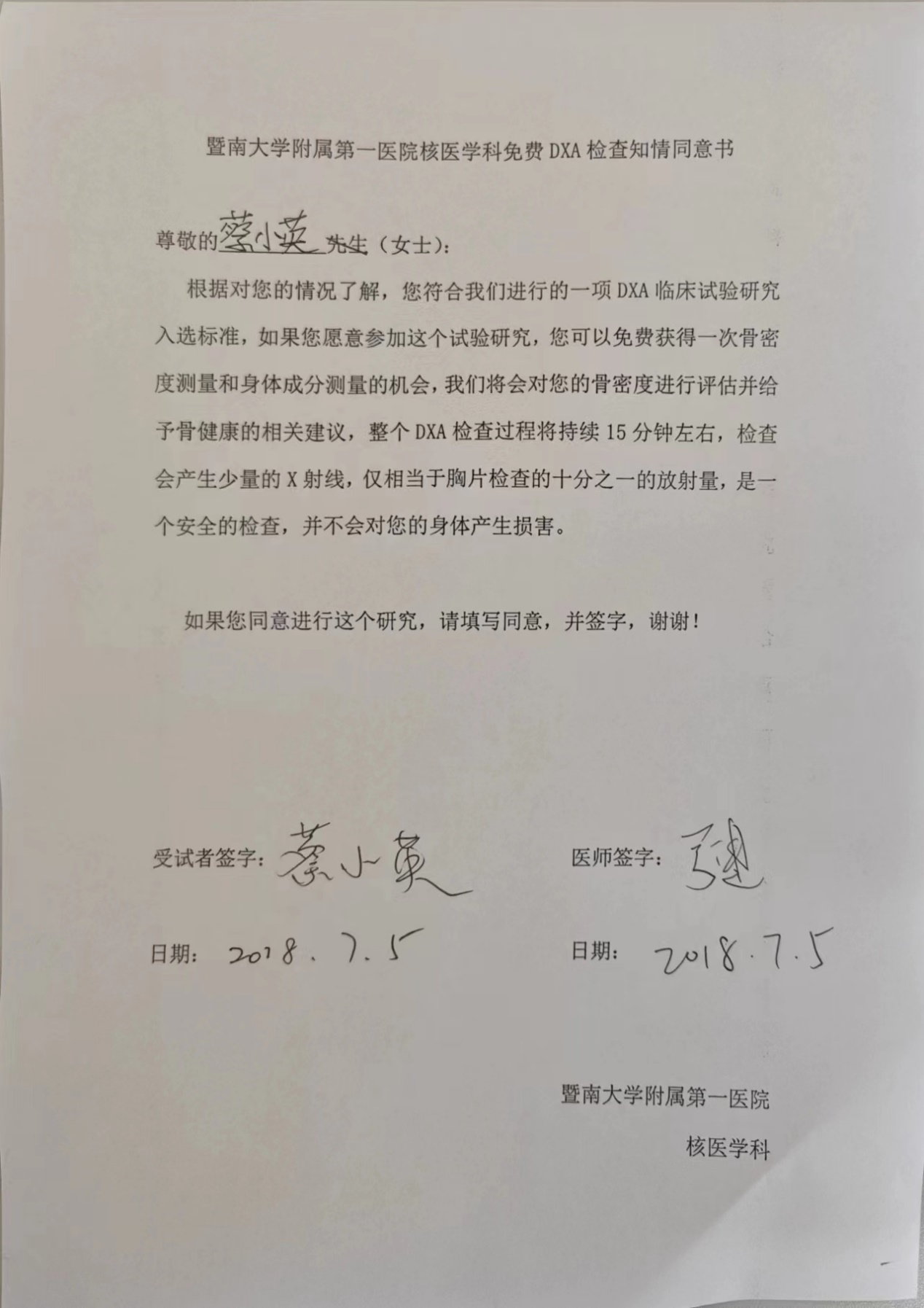


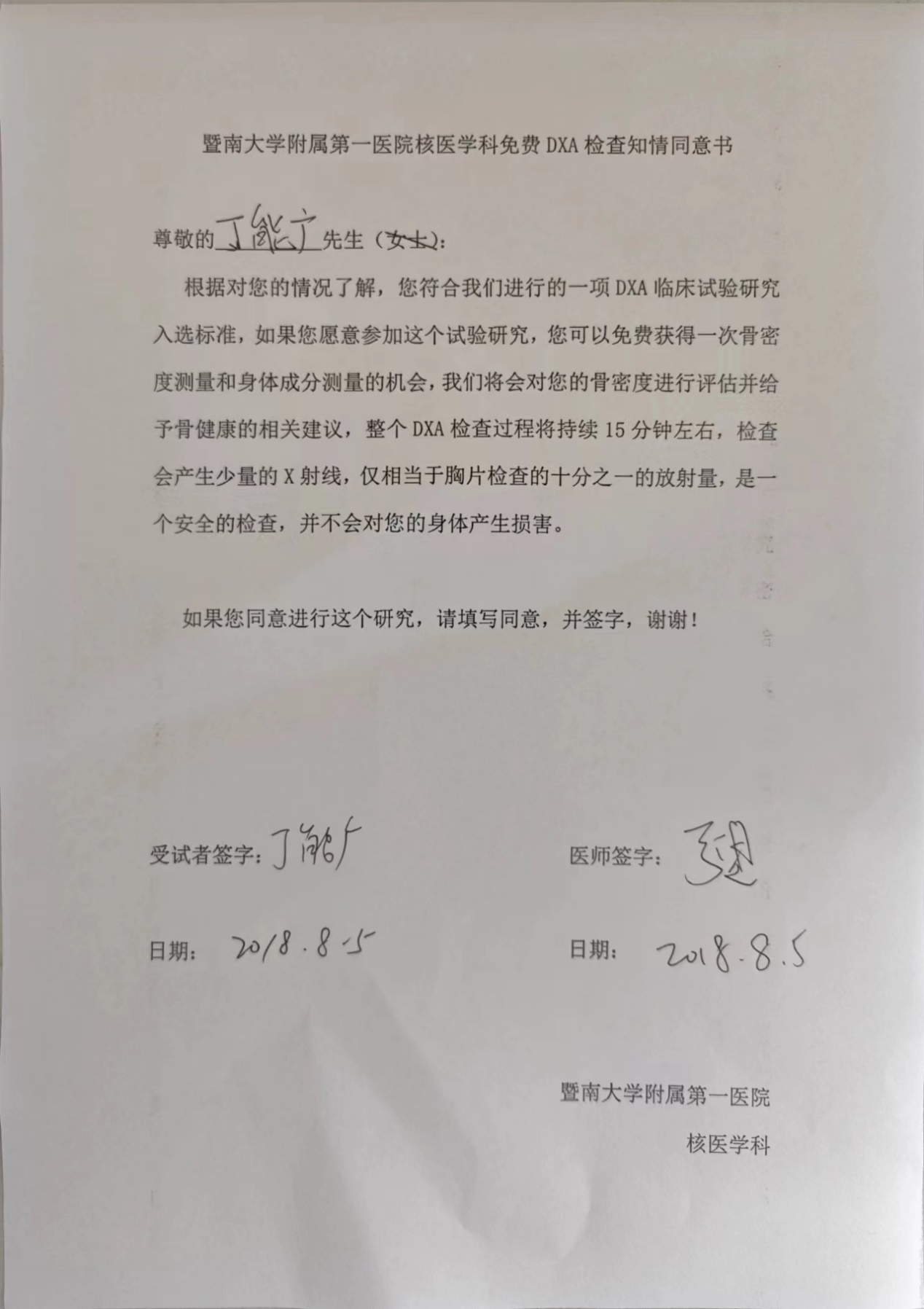


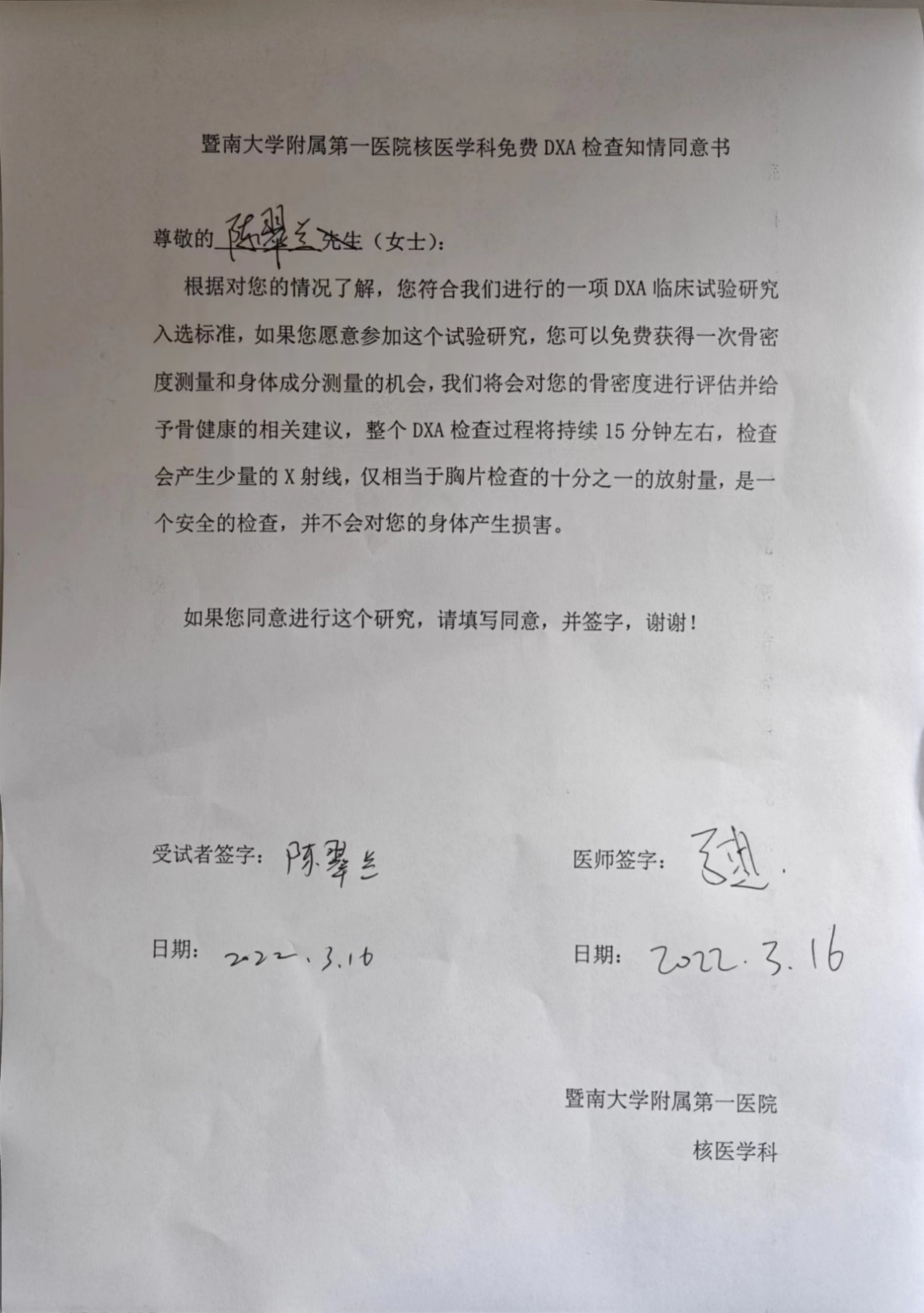


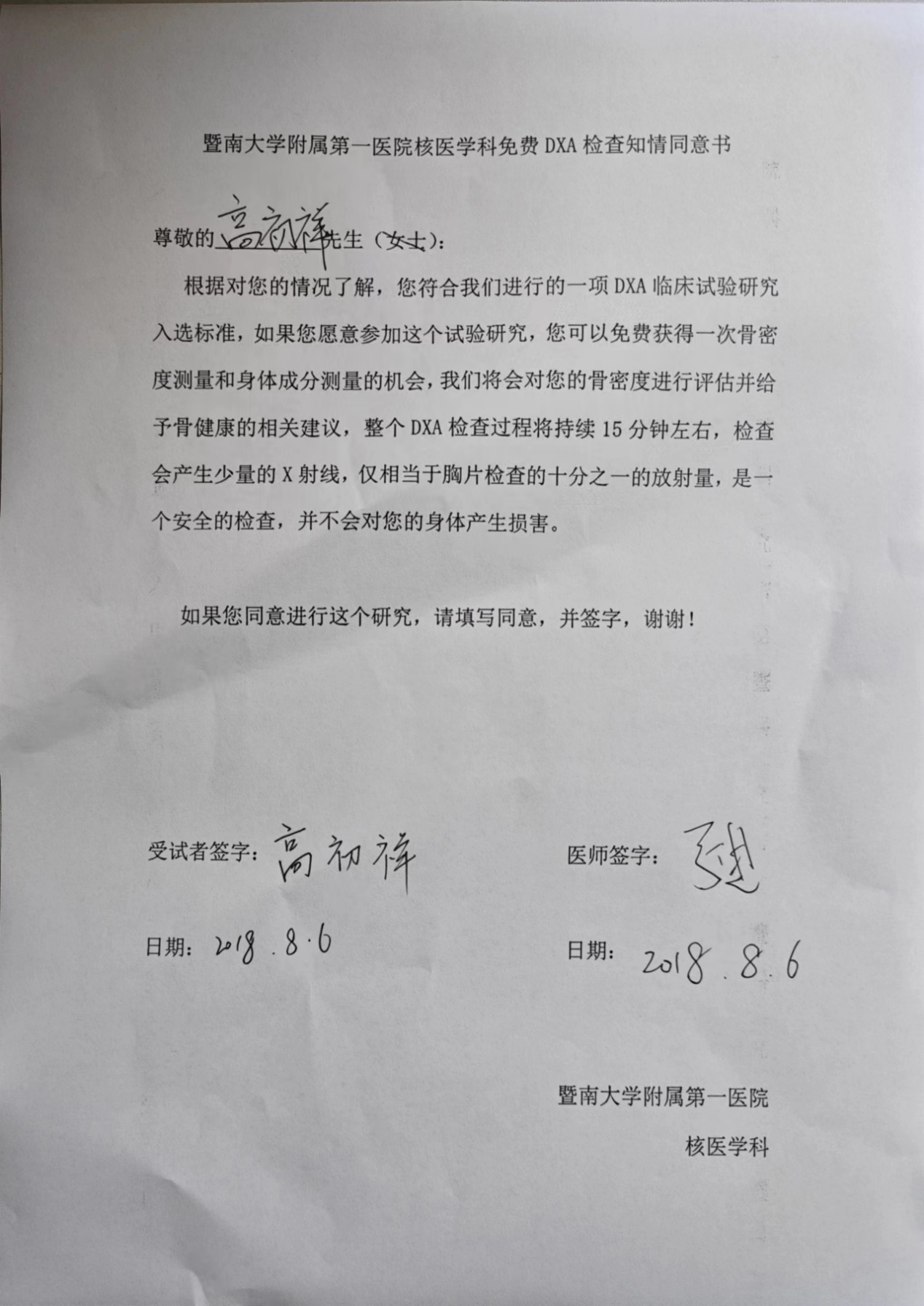


**Supplementary Table 1** Stepwise regression analysis for the prediction of TBF%, android %fat, and gynoid %fat in men in the equation group^a^

| Dependent DXA variable | Independent QCT variable | Prediction equation | Adjusted  R^2^ | SEE(also called RMSE) | Durbin-Watson | VIF | p (Shapiro-Wilk) | p (Breusch-Pagan) |
| --- | --- | --- | --- | --- | --- | --- | --- | --- |
| TBF% | T12/L1 %fat | 0.27×T12/L1 %fat+0.51×BMI+5.89 | 0.77 | 2.98 | 2.03 | 2.45 | 0.55 | 0.57 |
|  | L1/L2 %fat | 0.27×L1/L2 %fat+0.43×BMI+5.76 | 0.83 | 2.55 | 2.18 | 2.26 | 0.11 | 0.75 |
|  | L2/L3 %fat | 0.28×L2/L3 %fat+0.60×BMI-0.29 | 0.83 | 2.56 | 2.07 | 1.92 | 0.16 | 0.52 |
|  | L3/L4 %fat | 0.44×L3/L4 %fat+4.85 | 0.81 | 2.69 | 1.88 | 1.00 | 0.11 | 0.78 |
|  | L4/L5 %fat | 0.36×L4/L5 %fat+0.50×BMI-3.22 | 0.80 | 2.77 | 1.79 | 2.28 | 0.02 | 0.33 |
|  | L5/S1 %fat | 0.41×L5/S1 %fat+0.60×BMI-6.19 | 0.80 | 2.79 | 2.16 | 2.07 | 0.21 | 0.28 |
| Android %fat | T12/L1 %fat | 0.46×T12/L1 %fat+1.12×BMI-7.42 | 0.87 | 3.90 | 2.00 | 2.45 | 0.84 | 0.82 |
|  | L1/L2 %fat | 0.43×L1/L2 %fat+1.12×BMI-9.49 | 0.90 | 3.43 | 1.92 | 2.26 | 0.87 | 0.98 |
|  | L2/L3 %fat | 0.45×L2/L3 %fat+1.41×BMI-19.34 | 0.89 | 3.50 | 2.08 | 1.92 | 0.47 | 0.05 |
|  | L3/L4 %fat | 0.63×L3/L4 %fat+0.27×weight-14.86 | 0.89 | 3.50 | 1.85 | 1.98 | 0.65 | 0.28 |
|  | L4/L5 %fat | 0.58×L4/L5 %fat+1.22×BMI-23.85 | 0.87 | 3.83 | 1.84 | 2.28 | 0.82 | 0.95 |
|  | L5/S1 %fat | 16.54×log(L5/S1 %fat)+1.88×BMI-73.14 | 0.80 | 4.82 | 2.15 | 1.82 | 0.96 | 0.17 |
| Gynoid %fat | T12/L1 %fat | 0.13×T12/L1 %fat+0.59×BMI+7.40 | 0.47 | 3.68 | 2.05 | 2.45 | 0.01 | 0.23 |
|  | L1/L2 %fat | 0.21×L1/L2 %fat+16.90 | 0.52 | 3.50 | 2.14 | 1.00 | 0.12 | 0.60 |
|  | L2/L3 %fat | 0.18×L2/L3 %fat+0.46×BMI+6.42 | 0.56 | 3.32 | 2.17 | 1.92 | 0.01 | 0.39 |
|  | L3/L4 %fat | 0.31×L3/L4 %fat+10.29 | 0.59 | 3.23 | 2.18 | 1.00 | 0.04 | 0.35 |
|  | L4/L5 %fat | 0.31×L4/L5 %fat+9.61 | 0.56 | 3.34 | 2.20 | 1.00 | 0.15 | 0.24 |
|  | L5/S1 %fat | 0.40×L5/S1 %fat+6.91 | 0.65 | 2.98 | 2.58 | 1.00 | 0.03 | 0.09 |

DXA, dual-energy X-ray absorptiometry; adjusted R^2^, adjusted coefficient of determination; SEE, standard error of the estimate; TBF%, total body fat percentage; QCT, quantitative computed tomography. ^a^For details of the participants and procedures, see Table 1 and the text.

**Supplementary Table 2** Stepwise regression analysis for the prediction of TBF%, android %fat, and gynoid %fat in women in the equation group^a^

| Dependent DXA variable | Independent QCT variable | Prediction equation | Adjusted R^2^ | SEE(also called RMSE) | Durbin-Watson | VIF | p (Shapiro-Wilk) | | p (Breusch-Pagan) | |
| --- | --- | --- | --- | --- | --- | --- | --- | --- | --- | --- |
| TBF% | T12/L1 %fat | 0.34×T12/L1 %fat+24.36 | 0.68 | 3.54 | 2.23 | 1.00 | 0.09 | 0.86 | |  |
|  | L1/L2 %fat | 0.22×L1/L2 %fat+0.22×weight+12.37 | 0.64 | 3.74 | 2.48 | 1.79 | 0.06 | 0.61 | |  |
|  | L2/L3 %fat | 0.26×L2/L3 %fat+0.22×weight+9.62 | 0.71 | 3.36 | 2.31 | 1.55 | 0.14 | 0.81 | |  |
|  | L3/L4 %fat | 0.38×L3/L4 %fat+0.51×BMI+2.88 | 0.77 | 2.99 | 1.92 | 1.82 | 0.07 | 0.74 | |  |
|  | L4/L5 %fat | 0.50×L4/L5 %fat+0.57×BMI-7.41 | 0.80 | 2.83 | 2.39 | 1.63 | 0.84 | 0.56 | |  |
|  | L5/S1 %fat | 0.52×L5/S1 %fat+0.16×weight-3.52 | 0.81 | 2.73 | 2.45 | 1.66 | 0.96 | 0.50 | |  |
| Android %fat | T12/L1 %fat | 0.34×T12/L1 %fat+0.75×BMI+11.51 | 0.69 | 5.09 | 2.61 | 2.58 | 0.41 | 0.62 | |  |
|  | L1/L2 %fat | 0.34×L1/L2 %fat+0.31×weight+7.92 | 0.68 | 5.17 | 2.40 | 1.79 | 0.43 | 0.31 | |  |
|  | L2/L3 %fat | 0.47×L2/L3 %fat+0.27×BMI+1.53 | 0.83 | 3.83 | 2.53 | 1.55 | 0.59 | 0.79 | |  |
|  | L3/L4 %fat | 0.62×L3/L4 %fat+0.66×BMI-8.81 | 0.86 | 3.48 | 1.88 | 1.82 | 0.01 | 0.93 | |  |
|  | L4/L5 %fat | 0.75×L4/L5 %fat+0.84×BMI-23.88 | 0.84 | 3.69 | 2.29 | 1.63 | 0.85 | 0.36 | |  |
|  | L5/S1 %fat | 0.76×L5/S1 %fat+0.23×weight-17.14 | 0.83 | 3.82 | 2.05 | 1.66 | 0.67 | 0.46 | |  |
| Gynoid %fat | T12/L1 %fat | 0.21×T12/L1 %fat+31.45 | 0.30 | 4.83 | 1.90 | 1.00 | 0.04 | 0.13 | |  |
|  | L1/L2 %fat | 0.15×L1/L2%fat+0.47×BMI-0.13×age+27.77 | 0.32 | 4.78 | 2.25 | 1.23 | 0.07 | 0.31 | |  |
|  | L2/L3 %fat | 0.17×L2/L3 %fat+0.52×BMI-0.14×age+25.47 | 0.33 | 4.73 | 2.19 | 1.26 | 0.06 | 0.29 | |  |
|  | L3/L4 %fat | 0.31×L3/L4 %fat+21.76 | 0.31 | 4.81 | 1.62 | 1.00 | 0.24 | 0.24 | |  |
|  | L4/L5 %fat | 0.50×L4/L5 %fat-0.13×age+15.80 | 0.42 | 4.39 | 2.02 | 1.11 | 0.32 | 0.48 | |  |
|  | L5/S1 %fat | 0.51×L5/S1 %fat-0.12×age+15.08 | 0.51 | 4.04 | 2.21 | 1.07 | 0.15 | 0.34 | |  |

DXA, dual-energy X-ray absorptiometry; adjusted R^2^, adjusted coefficient of determination; SEE, standard error of the estimate; TBF%, total body fat percentage; QCT, quantitative computed tomography. ^a^For details of the participants and procedures, see Table 1 and the text.

**Supplementary Table 3** The correlation between average and difference values in Bland-Altman

| Average vs difference values | correlation coefficients |
| --- | --- |
| Figure 2a | -0.50* |
| Figure 2b | -0.46 |
| Figure 2c | -0.33 |
| Figure 2d | -0.25 |
| Figure 2e | **-0.24** |
| Figure 2f | **-0.27** |
| Figure 2g | **-0.62** |

**p* < 0.05. The bold data is Spearman’s correlation coefficients.

**Supplementary Table 4** Stepwise regression analysis for visceral adipose tissue related parameters

| Dependent variable | Independent variable | Prediction equation | Adjusted R^2^ | SEE(also called RMSE) | Durbin-Watson | VIF | p (Shapiro-Wilk) |
| --- | --- | --- | --- | --- | --- | --- | --- |
| male |  |  |  |  |  |  |  |
| QCT VAT | DXA VAT | 0.61×DXA VAT+71.91 | 0.86 | 134.77 | 1.56 | 1.00 | 0.16 |
| QCT VFV | DXA VFV | 1.66×DXA VFV+393.83 | 0.94 | 246.71 | 1.76 | 1.00 | 0.24 |
| female |  |  |  |  |  |  |  |
| QCT VAT | DXA VAT | 0.44×DXA VAT+186.35 | 0.70 | 163.04 | 2.29 | 1.00 | 0.26 |
| QCT VFV | DXA VFV | 1.26×DXA VFV+12.53×age+79.87 | 0.87 | 311.12 | 2.14 | 1.18 | 0.34 |

**Supplementary Table 5** Consistency test of visceral adipose tissue related parameters

| Dependent variable | Concordance correlation coefficient | Bland-Altman | |
| --- | --- | --- | --- |
|  |  | Mean difference | 95% LoA |
| male |  |  |  |
| QCT VAT vs DXA VAT | 0.70 | 293.74 | -190.92 to 778.41 |
| QCT VFV vs DXA VFV | 0.45 | -1049.50 | -1923.90 to -175.10 |
| female |  |  |  |
| QCT VAT vs DXA VAT | 0.58 | 268.57 | -427.87 to 965.00 |
| QCT VFV vs DXA VFV | 0.47 | -963.52 | -1737.62 to -189.41 |
