# Supplementary material for: Assessment of whole-body and regional body fat using abdominal quantitative computed tomography in Chinese women and men
Source: Lipids Health Dis. 2024 Feb 14;23:47. doi: 10.1186/s12944-024-02034-y (PMC10865662; doi:10.1186/s12944-024-02034-y)
Supplement: Supplementary file 2 — Additional file 2. [file 12944_2024_2034_MOESM2_ESM.docx]

Attached is the photos of the Chinese ethics review documentation, which are translated into English as follows:

IRB of the Fist Affiliated Hospital of Jinan University

Approval Letter

Batch number: KY-2022-079

| Project name | Based on quantitative CT technology to accompany you just DXA measurement of body composition research | | | | | | |
| --- | --- | --- | --- | --- | --- | --- | --- |
| Project leader | Hao Xu | | Application department | | | Department of nuclear medicine | |
| Research type | Clinical scientific research | | Research time | | | 2022.6-2023.6 | |
| Review document | 1. Application form for ethical review  2. Research scheme  3. Curriculum vitae of major researchers and division of labor of projects  4. Visa-free informed consent form | | | | | | |
| Review category | First trial | | Review mode | | | Expedited review | |
| Review date | 2022.6.2 | | Review location | | | No.1 conference hall | |
| Review results | Agree | agree with necessary amendments | | review with necessary amendments | disagree | | terminate or suspend the approved test |
|  | √ |  |  | |  | |  |
| term of validity | 2022.6.2-2023.6.2 | | | | | | |
| Review opinion  In accordance with the ethical principles of the Ministry of Health's measures for Ethical Review of Biomedical Research involving Human beings (2016), the SFDA Code for quality Management of Clinical Trials of drugs (2022), the provisions of Clinical Trials of Medical Devices (2022), the Helsinki Declaration of WMA and the International Ethical Guide for Human Biomedical Research, the contents and methods of the research have been reviewed by this Ethics Committee and approved. Please conduct clinical research / trials in strict accordance with the program and informed consent approved by the Ethics Committee to protect the health and rights of the subjects.  Chairman/Deputy Chairman:  year month date | | | | | | | |

Declaration: the composition and work of this Ethics Committee shall be strictly in accordance with the principles of China's GCP and ICH-GCP, the Helsinki Declaration and relevant laws and regulations, and its review and working process shall not be affected by any organization or individual outside the Ethics Committee.

Address of the Ethics Committee: 613 Huangpu Avenue West, Tianhe District, Guangzhou, Postal Code: 510632
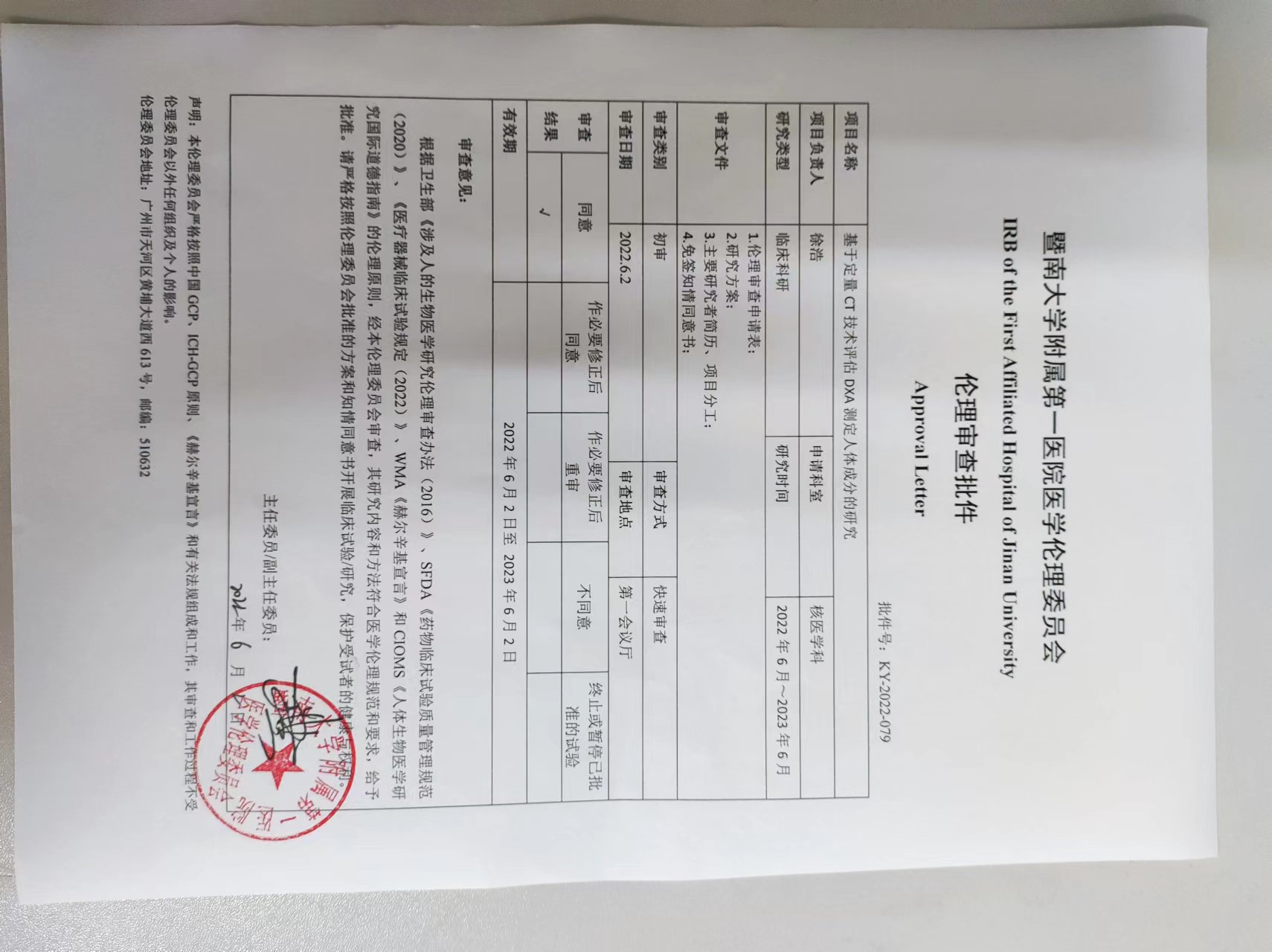


**Supplement Table 6** Physical characteristics and body composition of the participants, categorized according to sex

|  | Equation group | | Validation group | |
| --- | --- | --- | --- | --- |
|  | Males (n=50) | Females (n=50) | Males (n=18) | Females (n=21) |
|  | Mean ± SD/  Median(IQR) | Mean ± SD/  Median(IQR) | Mean ± SD/  Median(IQR) | Mean ± SD/  Median(IQR) |
| Age (yr) | 55.8±13.3 | 52.6±11.6 | 57.3±9.9 | 59.3±13.3 |
| Anthropometric measures | |  |  |  |
| Height (cm) | 167.1±5.7 | 157.8±4.9^a^ | 165.8±9.7^b^ | 155.2±6.4^ac^ |
| Weight (kg) | 63.3±10.2 | 55.0 (50.0-68.0) | 63.0±13.6 | 52.3±8.8^ac^ |
| BMI (kg/m^2^ ) | 22.6±2.9 | 23.4±3.7 | 22.8±3.3 | 21.6±3.1 |
| DXA measures | |  |  |  |
| DXA VAT mass (kg) | 0.9±0.5 | 0.6(0.4-1.1) | 0.9±0.5 | 0.7±0.4 |
| DXA VFV (cm^3^) | 977.1±567.8 | 658.6(398.0-1185.8) | 981.1±564.3 | 732.4±400.4 |
| TBF (kg) | 16.2±5.8 | 20.6(15.7-25.0)^a^ | 15.9±7.1^b^ | 1.5±0.7^ac^ |
| Android fat (kg) | 1.6±0.8 | 1.6(0.9-2.2)^a^ | 1.5±0.9^b^ | 1.5±0.7 |
| Gynoid fat (kg) | 2.3±0.8 | 3.3(2.6-4.2)^a^ | 2.2±0.9^b^ | 2.7±0.9^ac^ |
| QCT measures | |  |  |  |
| QCT VAT mass (kg) | 0.6(0.3-0.9) | 0.5(0.3-0.7) | 0.6±0.3 | 0.4±0.2 |
| QCT VFV (cm^3^) | 2046.6±972.3 | 1629.3(1236.0-2427.6) | 2090.1±1070.3 | 1572.4±654.4 |
| T12/L1 fat (kg) | 0.05(0.02-0.09) | 0.06(0.02-0.07) | 0.06±0.04 | 0.05(0.03-0.07) |
| L1/L2 fat (kg) | 0.06±0.04 | 0.07(0.04-0.11) | 0.06±0.04 | 0.06±0.04 |
| L2/L3 fat (kg) | 0.07±0.04 | 0.08(0.04-0.10)^a^ | 0.07±0.04 | 0.07±0.03 |
| L3/L4 fat (kg) | 0.07±0.04 | 0.08(0.05-0.10)^a^ | 0.07±0.04^b^ | 0.07±0.04^a^ |
| L4/L5 fat (kg) | 0.07±0.03 | 0.08(0.07-0.11)^a^ | 0.07±0.03^b^ | 0.07±0.03 |
| L5/S1 fat (kg) | 0.06±0.03 | 0.08(0.06-0.11)^a^ | 0.06±0.03^b^ | 0.07±0.03^ac^ |

BMI, body mass index; DXA, dual-energy X-ray absorptiometry; SD, standard deviation; IQR, interquartile range; VFV, visceral fat volume; VAT, visceral adipose tissue; TBF, total body fat; QCT, quantitative computed tomography. ^a^, *p* < 0.05 compared with participants in male equation group; ^b^, *p* < 0.05 compared with participants in female equation group; ^c^, *p* < 0.05 compared with participants in male validation group.

**Supplement Table 7** Pearson’s or Spearman’s correlation coefficients (r) for VAT mass, VFV, and fat measured using DXA or QCT

|  | males | Females |
| --- | --- | --- |
| DXA VAT mass vs QCT VAT mass | **0.92**** | **0.85**** |
| DXA VFV vs QCT VFV | 0.97** | **0.93**** |
| TBF vs T12/L1 fat | **0.89**** | **0.87**** |
| TBF vs L1/L2 fat | 0.90** | **0.87**** |
| TBF vs L2/L3 fat | 0.89** | **0.87**** |
| TBF vs L3/L4 fat | 0.90** | **0.86**** |
| TBF vs L4/L5 fat | 0.90** | **0.81**** |
| TBF vs L5/S1 fat | 0.88** | **0.86**** |
| Android fat vs T12/L1 fat | **0.91**** | **0.84**** |
| Android fat vs L1/L2 fat | 0.92** | **0.85**** |
| Android fat vs L2/L3 fat | 0.91** | **0.86**** |
| Android fat vs L3/L4 fat | 0.92** | **0.86**** |
| Android fat vs L4/L5 fat | 0.92** | **0.78**** |
| Android fat vs L5/S1 fat | 0.89** | **0.82**** |
| Gynoid fat vs T12/L1 fat | **0.77**** | **0.72*** |
| Gynoid fat vs L1/L2 fat | 0.78** | **0.71**** |
| Gynoid fat vs L2/L3 fat | 0.78** | **0.70**** |
| Gynoid fat vs L3/L4 fat | 0.79** | **0.71**** |
| Gynoid fat vs L4/L5 fat | 0.80** | **0.70**** |
| Gynoid fat vs L5/S1 fat | 0.81** | **0.76**** |

DXA, dual-energy X-ray absorptiometry; VFV, visceral fat volume; VAT, visceral adipose tissue; TBF, total body fat; QCT, quantitative computed tomography. **p* < 0.05, ***p* < 0.001. The bold data is Spearman’s correlation coefficients.

**Supplement Table 8** Stepwise regression analysis for the prediction of TBF, android fat, and gynoid fat in men in the equation group^a^

| Dependent DXA variable | Independent QCT variable | Prediction equation | Adjusted  R^2^ | SEE |
| --- | --- | --- | --- | --- |
| TBF | T12/L1 fat | 74.68×T12/L1 fat+0.97×BMI+0.11×height-27.67 | 0.89 | 1.94 |
|  | L1/L2 fat | 86.48×L1/L2 fat+0.23×weight-0.05×age-1.11 | 0.91 | 1.74 |
|  | L2/L3 fat | 74.69×L2/L3 fat+0.18×weight+0.45×BMI-10.41 | 0.91 | 1.75 |
|  | L3/L4 fat | 84.35×L3/L4 fat+0.26×weight-6.45 | 0.88 | 2.00 |
|  | L4/L5 fat | 92.81×L4/L5 fat+0.87×BMI-10.06 | 0.87 | 2.07 |
|  | L5/S1 fat | 95.51×L5/S1 fat+0.60×BMI+0.15×weight-12.40 | 0.89 | 1.89 |
| Android fat | T12/L1 fat | 13.07×T12/L1 fat+0.10×BMI-1.48 | 0.90 | 0.24 |
|  | L1/L2 fat | 12.07×L1/L2 fat+0.03×weight-1.14 | 0.92 | 0.22 |
|  | L2/L3 fat | 12.09×L2/L3 fat+0.03×weight-1.35 | 0.91 | 0.23 |
|  | L3/L4 fat | 13.22×L3/L4 fat+0.03×weight-1.18 | 0.89 | 0.26 |
|  | L4/L5 fat | 14.43×L4/L5 fat+0.09×BMI-1.57 | 0.88 | 0.27 |
|  | L5/S1 fat | 15.66×L5/S1 fat+0.12×BMI-2.05 | 0.88 | 0.26 |
| Gynoid fat | T12/L1 fat | 7.51×T12/L1 fat-0.01×age+0.13×BMI-0.57 | 0.75 | 0.38 |
|  | L1/L2 fat | 8.55×L1/L2 fa-0.01×age+0.11×BMI-0.10 | 0.77 | 0.36 |
|  | L2/L3 fat | 8.91×L2/L3 fat-0.01×age+0.11×BMI-0.28 | 0.78 | 0.35 |
|  | L3/L4 fat | 9.51×L3/L4 fat-0.01×age+0.10×BMI-0.13 | 0.76 | 0.37 |
|  | L4/L5 fat | 9.65×L4/L5 fat t-0.01×age+0.11×BMI-0.46 | 0.76 | 0.37 |
|  | L5/S1 fat | 12.93×L5/S1 fat-0.01×age+0.11×BMI-0.46 | 0.80 | 0.34 |

DXA, dual-energy X-ray absorptiometry; adjusted R^2^, adjusted coefficient of determination; SEE, standard error of the estimate; TBF, total body fat; QCT, quantitative computed tomography. ^a^For details of the participants and procedures, see Table 1 and the text.

**Supplement Table 9** Stepwise regression analysis for the prediction of TBF, android fat, and gynoid fat in women in the equation group^a^

| Dependent DXA variable | Independent QCT variable | Prediction equation | Adjusted R^2^ | SEE |
| --- | --- | --- | --- | --- |
| TBF | T12/L1 fat | 75.63×T12/L1 fat+0.41×weight-6.90 | 0.92 | 2.08 |
|  | L1/L2 fat | 70.01×L1/L2 fat+0.43×weight-8.60 | 0.92 | 2.2 |
|  | L2/L3 fat | 59.86×L2/L3 fat+0.46×weight-10.11 | 0.91 | 2.18 |
|  | L3/L4 fat | 59.56×L3/L4 fat+0.46×weight-10.20 | 0.91 | 2.22 |
|  | L4/L5 fat | 61.30×L4/L5 fat+0.46×weight-11.07 | 0.92 | 2.11 |
|  | L5/S1 fat | 70.72×L5/S1 fat+0.43×weight-10.30 | 0.93 | 2.02 |
| Android fat | T12/L1 fat | 11.53×T12/L1 fat+0.37×weight-0.99 | 0.86 | 0.33 |
|  | L1/L2 fat | 12.04×L1/L2 fat+0.04×weight-1.13 | 0.88 | 0.30 |
|  | L2/L3 fat | 11.18×L2/L3 fat+0.04×weight-1.32 | 0.89 | 0.29 |
|  | L3/L4 fat | 11.15×L3/L4 fat+0.04×weight-1.34 | 0.88 | 0.30 |
|  | L4/L5 fat | 8.69×L4/L5 fat+0.05×weight-1.67 | 0.83 | 0.35 |
|  | L5/S1 fat | 8.59×L5/S1 fat+0.05×weight-1.65 | 0.83 | 0.37 |
| Gynoid fat | L5/S1 fat | 8.36×L5/S1 fat-0.02×age+0.06×weight+0.07 | 0.74 | 0.56 |

DXA, dual-energy X-ray absorptiometry; adjusted R^2^, adjusted coefficient of determination; SEE, standard error of the estimate; TBF, total body fat; QCT, quantitative computed tomography. ^a^For details of the participants and procedures, see Table 1 and the text. Independent QCT variable (T12/L1 fat, L1/L2 fat, L2/L3, L3/L4, L4/L5) cannot establish the equation for predicting gynoid fat.

**Supplement Table 10** Summary of the Cross-validation of predictive equations developed in Table 3 and Table 4^a^

| Independent variable | Regression analysis | | |  | Bland–Altman | |
| --- | --- | --- | --- | --- | --- | --- |
|  | Intercept | Slope | Adjusted R^2^ | SEE | Mean difference | 95 LoA |
| Predicted TBF | | | | | | |
| Male (L1/L2) | -2.41 | 1.148 | 0.84 | 2.82 | -0.05 | -5.65 to 5.55 |
| Male (L2/L3) | -7.59 | 0.733 | 0.46 | 5.18 | -16.16 | -26.63 to -5.69 |
| Female (L5/S1) | -0.97 | 1.075 | 0.88 | 2.05 | 0.31 | -3.69 to 4.30 |
| Predicted Android fat | | | | | | |
| Male (L1/L2) | -0.07 | 1.050 | 0.83 | 0.36 | 0.00 | -0.69 to 0.70 |
| Female (L2/L3) | 0.06 | 0.944 | 0.86 | 0.26 | -0.03 | -0.53 to 0.47 |
| Predicted Gynoid fat | | | | | | |
| Male (L5/S1) | -0.35 | 1.176 | 0.71 | 0.50 | 0.04 | -0.95 to 1.02 |
| Female (L5/S1) | 0.08 | 1.016 | 0.68 | 0.51 | 0.12 | -0.86 to 1.10 |

DXA, dual-energy X-ray absorptiometry; adjusted R^2^, adjusted coefficient of determination; SEE, standard error of the estimate; TBF, total body fat; QCT, quantitative computed tomography; CI, confidence interval; LoA, limits of agreement. ^a^For details of the participants and procedures, see Table 1, Table 3, Table 4 and the text.
