# Supplementary figures and images for: Assessment of whole-body and regional body fat using abdominal quantitative computed tomography in Chinese women and men
Source: Lipids Health Dis. 2024 Feb 14;23:47. doi: 10.1186/s12944-024-02034-y (PMC10865662; doi:10.1186/s12944-024-02034-y)

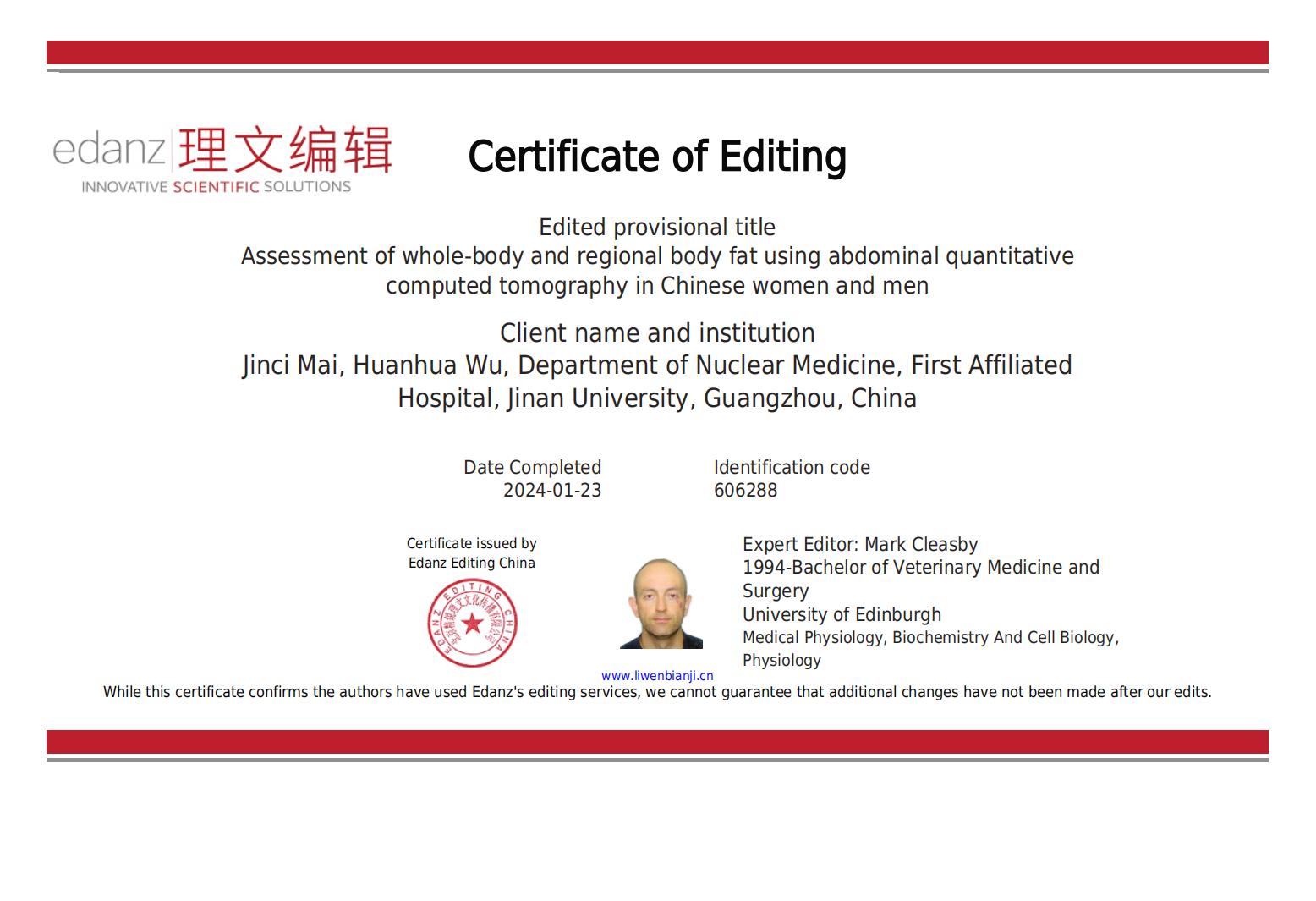

Supplement: Supplementary file 4 — Additional file 4. [file 12944_2024_2034_MOESM4_ESM.jpg]
